# The telomere-mitochondrial axis of aging in newborns

**DOI:** 10.18632/aging.203897

**Published:** 2022-02-15

**Authors:** Charlotte Van Der Stukken, Tim S. Nawrot, Rossella Alfano, Congrong Wang, Sabine A.S. Langie, Michelle Plusquin, Bram G. Janssen, Dries S. Martens

**Affiliations:** 1 Centre for Environmental Sciences, Hasselt University, Hasselt, Belgium; 2 Department of Public Health and Primary Care, University of Leuven, Leuven, Belgium

**Keywords:** telomere length, mitochondrial DNA content, p53, PGC-1α, aging

## Abstract

Aging starts at the beginning of life as evidenced by high variability in telomere length (TL) and mitochondrial DNA content (mtDNAc) at birth. Whether p53 and PGC-1α are connected to these age-related markers in early life is unclear. In this study, we hypothesized that these hallmarks of aging are associated at birth.

In 613 newborns from the ENVIRONAGE birth cohort, p53 and PGC-1α protein levels were measured in cord plasma, while TL and mtDNAc were measured in both cord blood and placental tissue. Cord blood methylation data of genes corresponding to the measured protein levels were available from the Human MethylationEPIC 850K BeadChip array. Pearson correlations and linear regression models were applied while accounting for selected covariates. In cord, a 10% increase in TL was associated with 5.22% (95% CI: 3.26 to 7.22; *p* < 0.0001) higher mtDNAc and −2.66% (95% CI: –5.04 to −0.23%; *p* = 0.032) lower p53 plasma level. In placenta, a 10% increase in TL was associated with 5.46% (95% CI: 3.82 to 7.13%; *p* < 0.0001) higher mtDNAc and −2.42% (95% CI: −4.29 to −0.52; *p* = 0.0098) lower p53 plasma level. Methylation level of TP53 was correlated with TL and mtDNAc in cord blood and with cord plasma p53 level.

Our study suggests that p53 may be an important factor both at the protein and methylation level for the telomere-mitochondrial axis of aging at birth.

## INTRODUCTION

Aging is universal, unavoidable, and starts at the very beginning of life with an acceleration at middle-age. The general cause of aging is considered the time-dependent accumulation of cellular damage [1–3]. The aging phenotype can be determined by cellular and molecular hallmarks that are generally considered to contribute to the aging process. Recently, primary hallmarks of aging were defined, which include genomic instability, telomere attrition, epigenetic alterations, loss of proteostasis, deregulated nutrient sensing, mitochondrial dysfunction, cellular senescence, stem cell exhaustion and altered intercellular communication [4].These pathways have mainly been studied individually. A major challenge is to dissect the interconnection between the candidate hallmarks and their contribution to the aging-phenotype.

In an experimental study using mouse embryonic fibroblasts, Sahin et al. [5] revealed a direct connection between two primary hallmarks of aging i.e., dysfunctional telomeres which resulted in altered mitochondrial biogenesis and function via the tumor suppressor *TP53*, which in turn repressed peroxisome proliferator-activated receptor gamma, coactivator 1 alpha and beta (*PGC1A* and *PGC1B*), also known as master regulators of the mitochondria. Through observations from their experimental study, the “core axis of aging” was put forth, involving telomeres, mitochondria, p53 and PGC.

Until now, research on aging-mechanisms has mainly been limited to experimental research. Translation of these findings to population-based studies is scarce and focusses mostly on the older segment of the population [6]. However, human aging may start early and even from birth onward. It is therefore important to extent research on aging-mechanisms to the younger segment of the population. We evaluated the connection between TL and mtDNAc in 613 newborns from the ENVIR*ON*AGE birth cohort and we evaluated whether p53 and PGC-1α are on the path of this aging biomarker link as experimentally suggested.

## METHODS

### Study population

Mothers with a singleton full-term birth were selected from the ongoing population-based prospective ENVIR*ON*AGE (ENVIRonmental influence ON early AGEing) birth cohort study, which is located in Limburg, Belgium. Detailed study procedures have been described previously [7]. Between 2010 and 2017, 1530 mother newborn pairs were recruited. In 691 samples, cord plasma protein levels (p53 and PGC-1α) were measured. After removing 4 missing data, 18 outlying data (3 SDs from the mean) for protein levels and 10 outlying data for TL and mtDNAc, a total of 613 participants were used for statistical analysis. After delivery, mothers completed study questionnaires to provide detailed information on maternal age, paternal age, maternal education, smoking status, parity, and newborn’s ethnicity. Maternal education was classified as “low” when mothers did not obtain any diploma, “middle” when they obtained a high school diploma, and “high” when they obtained a college or university diploma. Mothers were categorized as “never smoker”, “former smoker” when they had quit smoking before pregnancy, and “smoker” if they had smoked at any time point during pregnancy. Parity was categorized in mothers having their first newborn, having their second newborn, or having their third or more newborn. Newborns were classified as either “European” when two or more grandparents were European, or as “non-European” when at least three grandparents were of non-European origin. Other maternal and perinatal parameters such as maternal pre-pregnancy Body Mass Index (BMI), newborns’ sex, birth weight and birth date were collected from medical records after birth. Maternal pre-pregnancy BMI (kg/m²) was calculated based on data obtained during the first antenatal consultation. The date of conception was estimated by combining data on the first day of the mother’s last menstrual period and the first ultrasonographic examination. The ENVIR*ON*AGE study protocol has been conducted according to the Helsinki Declaration and was approved by the Ethical Committees of Hasselt University in Diepenbeek, Belgium (reference no. B371201216090 and B371201524537) and East-Limburg Hospital in Genk, Belgium. Written informed consent was provided by all participating mothers.

### Sample collection and preparation

Procedures for umbilical cord blood and placental tissue collection for TL and mtDNAc assessment have been described in detail previously [7]. BD Vacutainer^®^ Plus plastic whole-blood tubes with spray-coated K2EDTA (BD, Franklin Lakes, NJ, USA) were used to collect umbilical cord blood samples immediately after delivery. To obtain buffy coat and retrieve blood plasma, samples were centrifuged at 3,200 rpm for 15 min and stored separately. Within 10 minutes after delivery, placentas were collected and stored at –20°C. Four different placental biopsies (approximately 1–2 cm^3^) were taken at the fetal side at 4 cm from the umbilical cord and directly underneath the chorioamniotic membrane. Contamination by the chorioamniotic membrane was avoided by dissection followed by visual examination. All samples were stored at –80°C until DNA extraction. DNA was extracted using the QIAamp DNA Mini Kit (Qiagen Inc., Venlo, the Netherlands), according to the manufacturer’s instructions. The quantity and purity of the sample was measured with the Nanodrop spectrophotometer (ND-1000; Isogen Life Science, the Netherlands). DNA samples were normalized to ensure a uniform DNA input of 5 ng for each qPCR reaction, and this was checked using the Quant-iT™ PicoGreen^®^ dsDNA Assay Kit (Life Technologies, Europe). Extracted DNA was stored at –80°C until further use.

### Average relative TL and mtDNAc measurement

TL and mtDNAc were measured in cord blood buffy coat and placental tissue using a previously described modified quantitative real-time polymerase chain reaction (qPCR) protocol [8–10]. Details are provided in supplement (Supplementary Material). On each run, a 6-point serial dilution of pooled buffy coat or placental DNA was used to assess PCR efficiency as well as eight inter-run calibrators (IRCs) to account for inter-run variability. Non-template controls were also used in each run. All samples were measured in triplicate on a 7900HT Fast Real-Time PCR System (Applied Biosystems) in a 384-well format. qPCR curves for each sample were visually inspected and when technical problems were detected or triplicates showed too high variability, samples were removed for further analysis. Using qBase plus software (Biogazelle, Zwijnaarde, Belgium), all measurements were processed and normalized to a reference gene, taking into account run-to-run differences. Average relative TL was calculated by determining the ratio of one telomere gene copy number (T) to one reference gene (*36B4*) (S). Average mtDNAc was calculated by determining the average of the ratio of two mitochondrial gene copy numbers (*MTF3212/R3319* and *MT-ND1*) (M) to two nuclear reference genes (*36B4 and* *β-Actin*) (S). Telomere assay-reliability was assessed using an intra class correlation coefficient (ICC). The inter-assay ICC was 0.936 (95% CI: 0.808 to 0.969) and the intra-assay ICC was 0.952 (95% CI: 0.947 to 0.956).

### p53, PGC-1α and SIRT-1 protein measurement

After thawing cord blood plasma, 100 μl of plasma was quantified for p53 protein levels (U/mL) using a Human p53 ELISA Kit according to the manufacturer’s instructions (ref. ab46067, Abcam, Cambridge, United Kingdom). For PGC-1α (ng/mL), 100 μl of 1:250 diluted plasma was quantified according to the manufacturer’s instructions (ref. E-EL-H1359, Elabscience, Texas, USA). Two commercially available Human SIRT1 ELISA Kits were tested for the detection of cord plasma SIRT-1 protein levels, but for none of them the limit of detection was reached (ref. ab171573, Abcam, Cambridge, United Kingdom and ref. E-EL-H1546, Elabscience, Texas, USA). All protein levels were measured in duplicate using a FLUOstar Omega microplate reader (BMG Labtech, Ortenberg, Germany). To minimize variability, all samples were randomized across 96 well plates and all ELISAs for one protein were measured in one day, while using the same serial dilution. Five IRCs per plate were taken into account to control for potential variability between plates. The intra-assay coefficient of variation was 5% for p53, and 10% for PGC-1α while the inter-assay coefficient of variation reached 14.5% for p53 and 25% for PGC-1α.

### DNA methylation measurement

Cord blood DNA was used to determine the epigenome-wide DNA methylation levels. DNA samples were bisulfite-converted, amplified and then hybridized to the Illumina Infinium Human MethylationEPIC 850K BeadChip array (Illumina, San Diego, CA, USA) at the GenomeScan lab (Leiden, The Netherlands). The array measurements were scanned using an Illumina iScan and the data quality was assessed using the R script MethylAid. DNA methylation data preprocessing, quality control, outlier detection, batch effect removal and probe filtering were described previously [11]. In total, 57 CpG loci with their UCSC reference gene name referring to *TP53*, *PGC1A* or *SIRT1* were selected for the present study. Methylation levels of *TP53*, *PGC1A* and *SIRT1* were available for 205 participants and used for further analysis. Details are provided in Supplementary Table 1.

### Statistical analysis

All statistical analyses were performed using R studio version 3.6.2 (R Core Team, Vienna, Austria). Shapiro-Wilk’s test was used to check the normality of the distributions. Average relative TL, mtDNAc and cord plasma protein levels were log_10_-transformed to better approximate a normal distribution. For the descriptive statistics, continuous variables were presented as means ± standard deviation (SD) and categorical variables as numbers (frequency in percentage). Pearson correlation was used to systematically evaluate following correlations: (1) TL in cord blood and placenta and mtDNAc in the respective tissue, (2) TL in cord blood and placenta and cord plasma protein levels, (3) mtDNAc in cord blood and placenta and cord plasma protein levels and (4) cord plasma protein levels. Multiple linear regression was applied to further confirm the associations independent of potential confounding effects. We adjusted for a priori selected covariates based on known associations of these factors with TL, mtDNAc, and protein levels as shown previously in multiple studies [8, 12]: Technical covariates (sample storage and batch effects), newborn’s sex, gestational age, maternal BMI, maternal and paternal age, ethnicity, parity, smoke status, maternal education and month of delivery. All model estimates were presented as percentage difference with 95% CI and expressed for a 10% increment in explanatory variable.

In a secondary explorative analysis, we evaluated in a subpopulation whether cord blood methylation levels of genes corresponding to our measured protein levels (p53, PGC-1α and SIRT1) were related to the studied age-related markers and protein levels, to further explore the interrelationship of the telomere-mitochondrial aging axis. We restricted these DNA methylation association studies to age-related markers measured in cord blood, as it is known that DNA methylation levels are highly tissue- and cell type- specific [13–15]. Since multiple CpGs were measured for each gene, we first made a correlation matrix showing the Pearson correlations between the CpGs for each gene. Second, we performed Principal Component Analysis (PCA) to reduce the dimensionality of our dataset [16]. To determine the number of Principal components (PCs) to retain in our analysis, we created scree plots for each gene (Supplementary Figure 1). PCs on the left side of the “elbow” of the graph were retained in the analysis. For all genes, this resulted in 5 PCs to retain. Next, we correlated the PCs with the CpGs, to determine their loadings (Supplementary Table 2). CpG loci were selected as relevant to a factor if the absolute value of their factor loadings were larger than 0.45 [16]. First, these first five PCs were used to assess the Pearson correlations between methylation data and both cord blood age-related markers and cord plasma protein levels. Second, multiple linear regression was applied to confirm these associations while adjusting for the aforementioned covariates.

## RESULTS

### Study population characteristics

Demographic, pregnancy-related and perinatal characteristics of the mother-newborn pairs included in this study (*n* = 613) were summarized in Table 1. Our study subset was representative for the original study population (Supplementary Table 3). Mothers were on average 29.3 (SD: 4.6) years old and had an average pre-pregnancy BMI of 24.6 (SD: 4.8) kg/m^2^. Half of the participating women were highly educated (50%). The majority of the pregnant women never smoked cigarettes (63%), 25% stopped smoking before pregnancy, and 11% kept smoking on the average 3.4 cigarettes/day during pregnancy. Among the newborns, 52% were girls. Newborns had an average gestational age of 39.2 (SD: 1.67) weeks, an average birth weight of 3420 (SD: 496) grams, and most were of European origin (86%). Information about the age-related and protein markers is given in Table 2. Cord plasma SIRT-1 levels were excluded from the analysis since the measurements did not reach the limit of detection (LOD) of 0.31−0.63 ng/ml.

**Table 1:** Descriptive characteristics of mother-newborn pairs from a subset (*n* = 613) of the ENVIR*ON*AGE birth cohort.

**Characteristic**	**Mean ± SD or *n* (%) (*n* = 613)**
**Mothers**	
Age, y	29.3 ± 4.6
Pre-pregnancy BMI, kg/m^2^	24.6 ± 4.8
Educational level	
Low	79 (12.9%)
Middle	227 (37.0%)
High	307 (50.1%)
Smoking status	
Never smoker	391 (63.8%)
Former smoker	154 (25.1%)
Current smoker	68 (11.1%)
Parity	
1	337 (55.0%)
2	206 (33.6%)
≥3	70 (11.4%)
**Newborns**	
Sex	
Female	321 (52.4%)
Gestational age, wk	39.2 ± 1.7
Birth weight, g	3420 ± 496
Ethnicity	
European-Caucasian	533 (86.9%)
Season of birth	
Winter	150 (24.5%)
Spring	149 (24.3%)
Summer	151 (24.6%)
Autumn	163 (26.6%)

**Table 2:** Age-related markers measured in a subset (*n* = 613) of the ENVIR*ON*AGE birth cohort.

**Age-related marker**	**Mean ± SD**
**Placenta**	
TL (T/S ratio)	0.99 ± 0.26
mtDNAc (M/S ratio)	1.05 ± 0.65
**Cord blood**	
TL (T/S ratio)	0.99 ± 0.19
mtDNAc (M/S ratio)	1.03 ± 0.57
p53 plasma level (U/ml)	12.5 ± 9.72
PGC-1α plasma level (μg/ml)	1145 ± 360

Data presented as mean ± SD or number of participants (%). TL and mtDNAc are normalized separately in cord blood and placental tissue. Abbreviations: TL: telomere length; mtDNAc: Mitochondrial DNA content.

### The telomere-mitochondrial axis of aging: links between TL, mtDNAc, p53 and PGC-1α

#### Unadjusted correlations

Cord blood and placental TL were positively correlated (r = 0.40, *p* < 0.0001, Supplementary Figure 2A), but no correlation between cord blood and placental mtDNAc was observed (Supplementary Figure 2B). The following correlations were systematically evaluated: (1) TL and mtDNAc, (2) TL and cord plasma protein levels, (3) mtDNAc and cord plasma protein levels and (4) between the cord plasma protein levels.

First, a positive correlation was found between TL and mtDNAc in both cord blood (r = 0.23, *p* < 0.0001, Supplementary Figure 3A) and placental tissue (r = 0.28, *p* < 0.0001, Supplementary Figure 3B).

Second, cord plasma p53 levels were negatively correlated with cord blood TL (r = −0.13, *p* = 0.0015, Supplementary Figure 4A) but not with placental TL (Supplementary Figure 4B). Cord plasma PGC-1α levels were negatively correlated with both cord blood TL (r = −0.10, *p* = 0.013, Supplementary Figure 4C) and placental TL (r = −0.09, *p* = 0.029, Supplementary Figure 4D).

Third, no correlation was observed between cord plasma p53 levels and cord blood mtDNAc (Supplementary Figure 5A), but tended to be negatively correlated with mtDNAc in placenta (r = −0.07, *p* = 0.094, Supplementary Figure 5B). No correlation was observed between cord plasma PGC-1α levels and cord blood mtDNAc (Supplementary Figure 5C), but a negative correlation was observed with placental mtDNAc (r = −0.11, *p* = 0.0082, Supplementary Figure 5D).

**Figure 1:**
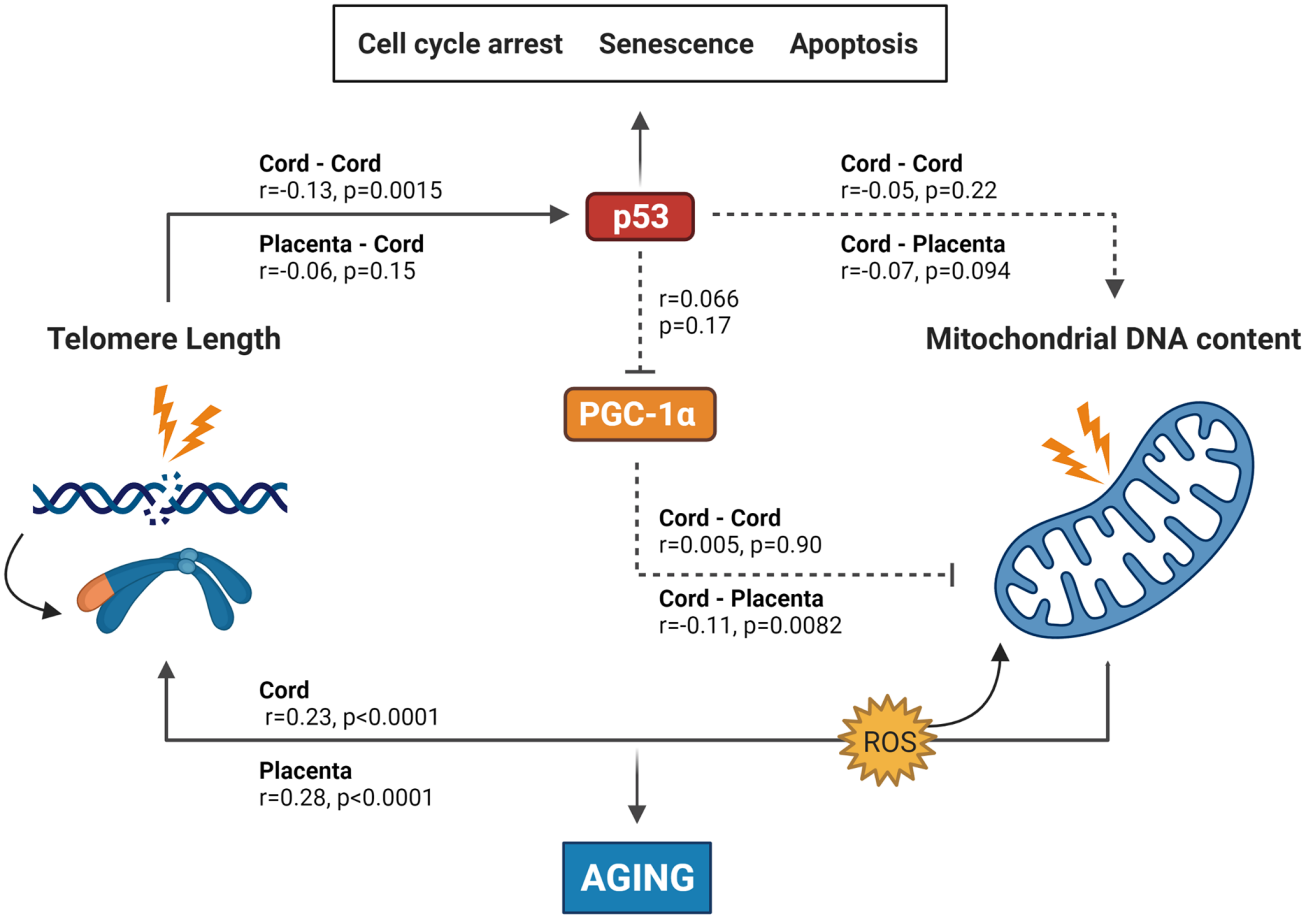
**Summary of the results found in this study integrated into the experimentally based telomere-mitochondrial axis of biological aging hypothesis.** DNA damage and telomere shortening activate p53 leading to growth arrest, senescence or apoptosis. p53 might also impair mitochondrial function and mitochondrial DNA content indirectly through suppression of PGC-1α – one of the master regulators of the mitochondria – leading to mitochondrial comprise and increased ROS levels, which leads to more DNA damage including telomere shortening. p53 and PGC-1α could therefore be central players in the association between telomere length and mitochondrial DNA content and subsequently in the aging process. Solid lines represent significant associations between age-related or protein markers, while non-significant associations are represented by dotted lines. p53 and PGC-1α levels were only measured in cord blood, while TL and mtDNAc were measured in both cord blood and placental tissue. Abbreviations: p53: tumor suppressor protein 53; PGC-1α: peroxisome proliferator-activated receptor gamma co-activator 1 alpha protein. Figure based on the experimental work of Sahin et al. [5, 33].

Fourth, cord plasma p53 and PGC-1α levels were not correlated (Supplementary Figure 6). In Figure 1, a visual summary of all correlations is presented, which is based on the experimentally derived telomere-mitochondrial axis of aging hypothesis.

#### Covariate adjusted linear models

Using adjusted regression models, we further evaluated the associations between TL and (1) mtDNAc and (2) cord plasma protein levels (Table 3). We furthermore evaluated the associations between mtDNAc and the cord plasma protein levels, and also the association between the cord plasma protein levels (Supplementary Table 4). All associations were adjusted for technical covariates (sample storage and batch effects), newborn’s sex, gestational age, maternal BMI, maternal and paternal age, ethnicity, parity, smoke status, maternal education and month of delivery. First, an increase in cord blood and placental TL was associated with higher mtDNAc in the respective tissue. A 10% increase in cord blood TL was associated with 5.22% (95% CI: 3.26 to 7.22; *p* < 0.0001) higher cord blood mtDNAc and a 10% increase in placental TL was associated with 5.46% (95% CI: 3.82 to 7.13%; *p* < 0.0001) higher placental mtDNAc. Second, both cord blood and placental TL were associated with lower p53 levels, which is largely in line with the observed unadjusted correlations. A 10% increase in cord blood TL was associated with −2.66% (95% CI: −5.04 to −0.23%; *p* = 0.032) lower p53 plasma levels, while a 10% increase in placental TL was associated with –2.42% (95% CI: −4.29 to −0.52; *p* = 0.0098) lower p53 plasma levels. The observed correlations between TL and cord blood PGC-1α levels could not be confirmed in adjusted models. Furthermore, the correlations observed between mtDNAc and the protein levels, nor between the protein levels themselves could not be confirmed in full adjusted regression models (Supplementary Table 4).

**Table 3:** Association between TL in cord blood and placenta and (1) mtDNAc in the respective tissue and (2) cord plasma protein levels in the telomere-mitochondrial axis of aging.

	**Cord TL (*n* = 603)**		**Placenta TL (*n* = 558)**	
	**% difference (95% CI)**	***P*-value**	**% difference (95% CI)**	***P*-value**
**Cord mtDNAc**	5.22 (3.26, 7.22)	<0.0001	–0.45 (–1.96, 1.08)	0.56
**Placenta mtDNAc**	1.02 (–1.06, 3.16)	0.34	5.46 (3.82, 7.13)	<0.0001
**Cord p53**	–2.66 (–5.04, –0.23)	0.032	–2.42 (–4.29, –0.52)	0.0098
**Cord PGC-1α**	0.17 (–0.85, 1.21)	0.74	0.49 (–0.31, 1.29)	0.22

Estimates are presented as percentage difference with 95% CI for a 10% change in explanatory variable. All models are adjusted for technical covariates (sample storage and batch effects), newborn’s sex, gestational age, maternal BMI, maternal and paternal age, ethnicity, parity, smoke status, maternal education and month of delivery. Cord blood and placental Telomere Length represent the response variables, while the markers in the first column represent the exposure variables. Abbreviations: TL: telomere length; mtDNAc: mitochondrial DNA content.

### Cord blood *TP53*, *PGC1A* and *SIRT1* methylation levels and the cord blood telomere-mitochondrial axis of aging

#### Unadjusted correlations

First, a Pearson correlation heatmap of the methylation levels of all CpGs for each gene (*TP53*, *PGC1A* and *SIRT1*, separately) is shown in Supplementary Figure 7. Second, the unadjusted Pearson correlation matrix between *TP53*, *PGC1A* and *SIRT1* methylation data (reflected by 5 PCs) and both cord blood age-related markers and protein levels in a subset of our study data (*n* = 205) is shown in Supplementary Figure 8. The first five PCs cumulatively explained 41%, 58% and 59% of the variation of *TP53*, *PGC1A* and *SIRT1,* respectively. In Supplementary Figures 9 and 10, significant correlations were additionally presented in correlations plots.

For *TP53* methylation data, PC1 was negatively correlated with cord plasma p53 level (r = −0.17, *p* = 0.038), PC2 was positively correlated with cord blood TL (r = 0.20, *p* = 0.010) and PC5 was negatively correlated with cord blood mtDNAc (r = −0.16, *p* = 0.00073). The most relevant CpG to PC1 based on factor loadings (cg12041429, Supplementary Table 2) was located within the 5′ untranslated region (5′UTR), meaning between the transcription start site (TSS) and the translation initiation codon (ATG) (Supplementary Table 1). The most relevant CpG for PC2 (cg18198734, Supplementary Table 2), was located in the gene body, meaning between the ATG and stop codon; irrespective of the presence of introns, exons, TSS or promoters (Supplementary Table 1). For PC5, absolute values of CpG factor loadings were not larger than 0.45, therefore, no CpG loci were selected as relevant to this factor.

For *PGC1A* methylation data, PC3 was positively correlated with cord blood TL (r = 0.18, *p* = 0.013). However, no CpG loci were selected as relevant to this factor.

For SIRT1 methylation data, no significant correlations were found with any of the measured markers.

#### Covariate adjusted linear models

The associations between *TP53*, *PGC1A* and *SIRT1* methylation data and cord blood age-related markers and cord plasma protein levels were further evaluated using regression models. All models were adjusted for technical covariates (sample storage and batch effects), newborn’s sex, gestational age, maternal BMI, maternal and paternal age, ethnicity, parity, smoke status, maternal education and month of delivery (Supplementary Table 5). The observed unadjusted correlations between methylation levels and both cord blood age-related markers and cord plasma protein levels could be confirmed after adjustment.

## DISCUSSION

Aging is a complex universal and unavoidable physiological phenomenon. Both telomere attrition and mitochondrial damage are main factors in this biological process, but have mostly been studied independently. We [9, 17] and others [18–20] already observed large variability in TL and mtDNAc among newborns and we found that TL at birth predicts later life TL [17]. In the current study with 613 mother-newborn pairs, we evaluated in cord blood and placenta if TL is connected to mtDNAc, whether p53 and PGC-1α are connected to these age-related markers and whether we may confirm the core axis of aging hypothesis in newborns.

Our study has two main findings: First, TL in cord blood and placenta is positively associated with mtDNAc in the respective tissue. Second, TL in cord blood and placenta is negatively associated with cord plasma p53 level, and the TL connection with p53 regulation is furthermore strengthened with the observed association with *TP53* methylation levels. These findings were confirmed after adjustment for potential confounding factors, and are in line with the proposed hypothesis as displayed in Figure 1. Our results partly confirmed the contribution of p53 in the telomere-mitochondrial axis of aging in early-life, however, the connection between p53 and mtDNAc and between the protein levels remains unconfirmed in our study.

Negative correlations between placental mtDNAc and both p53 and PGC1-α protein levels were found, but did not survive full adjustment for potential confounders. Also, p53 and PGC1-α were not correlated. However, methylation levels of the genes corresponding to the measured proteins show correlations with all markers that are in line with our hypothesis. As for the methylation levels, the highest contributing CpG loci were either located in the 5′UTR or in the gene body. In the 5′UTR, essential promotor elements are located and methylation of these promotor sequences will downregulate methylation, while gene-body methylation has been observed to be positively correlated with gene expression levels [21].

There was furthermore a strong positive association between cord blood and placental TL, but not between cord blood and placental mtDNAc, which may indicate that tissue-specific differences in mtDNAc are larger than tissue-specific differences in TL. This finding is in line with observations that TLs are highly correlated in different tissues within the same individual [17, 22]. For mtDNAc, the difference largely depends on the difference in energy demand of each cell type [23].

How can our observations be explained based on the proposed hypothesis (Figure 1)? DNA damage and excessive telomere shortening caused by intracellular stresses or environmental signals activate p53 [12, 24], which has commonly been referred to as the ‘guardian of the genome’ due to its role in protecting the cell from DNA damage and acting as a central hub in many biological downstream pathways [25, 26]. It regulates the expression of a variety of genes involved in different cellular functions, including cell-cycle regulation, apoptosis, DNA replication and repair, cell proliferation, cellular stress response and negative regulation of p53 [27]. Its activation modulates cellular senescence and organismal aging [28], whereas loss or mutation within the *TP53* gene (which encodes p53) prevent cell death and increase cancer risk [29]. In this model, activated p53 directly suppresses PGC-1α, which alters the mitochondria (decreased mitochondrial function, impaired ATP generation and increased reactive oxygen species (ROS) production) which can lead to accelerated biological aging. Contradictory, there is growing evidence that p53 helps maintain the mitochondrial genome through translocation into mitochondria and interactions with mtDNA repair proteins. Park et al. [30] suggest that in unstressed cells, p53 functions as mito-checkpoint protein and regulates mtDNA copy number and mitochondrial biogenesis. Conversely, stress activated p53 (through DNA damage or telomere shortening) results in impaired mitochondrial biogenesis [30].

The findings of our study are supported by both experimental and population-based human studies. First of all, the link between telomeres and mitochondria was initially proposed in the experimental study of Sahin et al. [31], where telomerase-deficient mice (with a high level of dysfunctional telomeres) showed a strong activation of the *TP53* gene, which resulted in suppression of *PGC1A* and *PGC1B* genes – the master regulators of mitochondrial biogenesis and metabolism – leading to comprised mitochondrial biogenesis. The link between these age-related markers was also made in several human studies [6, 32]. In a community sample of 392 healthy adults, Tyrka et al. [32] showed a positive correlation between leukocyte TL and mtDNAc, but the underlying mechanism remained undetermined. In 166 elderly, an association has been demonstrated between leukocyte TL and mtDNAc with *SIRT1* as a key role player in the telomere-mitochondrial interactome [6]. *SIRT1* expression was also shown to be inversely associated with *TP53* expression, which subsequently altered the expression of *PGC1A* [6].

What is the importance of our findings? Excessive telomere shortening to a critical length (Hayflick limit) [9] can lead to genome instability and senescence. It is also associated with an increased risk of age-related diseases, such as cardiovascular diseases [10], metabolic diseases [11] and cancer [12]. Mitochondria are the biochemical power plants of eukaryotic cells. Compared with nuclear DNA (nDNA), mtDNA is more vulnerable to damage induced by endogenous and exogenous agents. This due to the lack of protective histones, the small mitochondrial genome, its close proximity to the respiratory chain and its limited DNA repair system [15]. Mitochondrial damage might result in metabolic changes such as impaired ATP generation, increased production of ROS and decreased levels of ROS-detoxifying enzymes, which cause additional genomic instability as observed in aging and cancer [16]. By unraveling the mechanisms underlying the association between age-related markers in an early life context, we can further investigate how these important regulators may be influenced by early life exposures, and how this may lead to vulnerability for disease in later life. In addition, more research is needed to determine whether tracking and fixed ranking of TL among newborns, as evidence by Martens et al. [17], can be explained by variations of key-regulator levels in the core axis of aging. By strengthening our knowledge about tracking and ranking mechanisms involved in the complete axis of aging, improved measures can be taken to promote healthy aging across the life course.

Our study has several strengths. First of all, this is to our knowledge the first study that has investigated the key regulators of the telomere-mitochondrial axis of aging at birth. Second, we used a relatively large sample size (*n* = 613) within the ENVIR*ON*AGE birth cohort, which is representative for the ENVIR*ON*AGE birth cohort at large [7]. The third strength is the availability of different biological matrices (placenta, cord blood and cord plasma), in which the age-related markers were measured over different biological levels (TL, mtDNAc, proteins levels and methylation levels). This made it possible to investigate the telomere-mitochondrial axis of aging independent of the biological matrix.

Besides our strengths, we also had to deal with some limitations. First, in addition to p53 and PGC-1α, SIRT-1 is also considered to be a major contributor in the telomere-mitochondrial axis of aging pathway [6]. Unfortunately, this could not be confirmed in our study, since were not able to detect cord plasma SIRT-1 levels above the limit of detection of several commercially available ELISA kits. As evidenced by other studies [6, 33], SIRT-1 plays a role in the molecular axis of aging and might provide interesting information to confirm our hypothesis. A second limitation is that p53 and PGC-1α were only measured in cord plasma but not in placental tissue, due to incompatibility with the measurement assays and due to limited placental tissue availability. As the aging process may be different for different tissues, the availability of placental protein levels of p53 and PGC-1α would provide more insight in the placental aging processes, this especially as placental tissue is a temporary end of life organ. Third, other non-studied markers playing a role in the telomere-mitochondrial axis of aging were not investigated and we can therefore not exclude that our findings are influenced by other important gene regulators. Fourth, our results only give an indication that p53 protein and methylation is linked with TL and mtDNAc.

In conclusion, we show that TL is connected to mtDNAc and that epigenetic and protein differences related to p53 might be involved in connecting these age-related markers at birth. This might be in line with the experimentally proposed telomere-mitochondrial axis of aging and gains important insight into the early life aging process.

## Supplementary Materials

Supplementary Methods

Supplementary Figures

Supplementary Tables
