# Supplementary material for: The telomere-mitochondrial axis of aging in newborns
Source: Aging (Albany NY). 2022 Feb 15;14(4):1627–50. doi: 10.18632/aging.203897 (PMC8908926; doi:10.18632/aging.203897)
Supplement: Supplementary Methods [file aging-14-203897-s001.pdf]

SUPPLEMENTARY METHODS

Reaction mixtures and PCR cycling conditions for telomere length (TL) and mitochondrial DNA content (mtDNAc) measurements

The master mix for TL measurement contained 1× QuantiTect SYBR Green PCR master mix (Qiagen, Inc., Venlo, the Netherlands), 2 mM dithiothreitol (DTT), 300 nM telg primer (ACACTAAGGTTTGGGTTTGGGTT TGGGTTTGGGTTAGTGT) and 900 nM telc primer (TGTTAGGTATCCCTATCCCTATCCCTATCCCTAT CCCTAACA). Used cycling conditions were: 1 cycle at 95°C for 10 min, followed by 2 cycles at 94°C for 15 sec and 49°C for 2 min and 30 cycles at 94°C for 15 sec, 62°C for 20 sec, and 74°C for 1 min and 20 sec. The single-copy gene reaction mixture contained 1× QuantiTect SYBR Green PCR master mix, 300 nM 36B4u primer (CAGCAAGTGGGAAGGTGTAATCC) and 500 nM 36B4d primer (CCCATTCTATCAT CAACGGGTACAA). Used cycling conditions were: 1 cycle at 95°C for 10 min, followed by 40 cycles at 95°C for 15 sec, and 58°C for 1 min and 20 sec [1].

The master mix for mtDNAc measurement consisted of Fast SYBR® Green I dye 2× (Applied Biosystems; 5 µL/reaction), 300 nm forward (5′-CACCCAAGAA CAGGGTTTGT-3′ for MTF3212/3319, and 5′-ATGG CCAACCTCCTACTCCT-3′ for MT-ND1) and 300 nm reverse primer (5′-TGGCCATGGGTATGTTGTTAA-3′ for MTF3212/3319 and 5′-CTACAACGTTGGGGCC TTT-3′ for MT-ND1) and RNase free water (1.9 µL/ reaction). thermal cycling profile was the same for all transcripts: 20 sec at 95°C for activation of the AmpliTaq Gold® DNA-polymerase, followed by 40 cycles of 1 sec at 95°C for denaturation and 20 sec at 60°C for annealing/extension. Amplification specificity and absence of primer dimers was confirmed by melting curve analysis at the end of each run. After thermal cycling, raw data were collected and processed. CT (cycle threshold)–values of the two mitochondrial genes were normalized relative to the nuclear reference genes according to the qBase software (Biogazelle, Zwijnaarde, Belgium). The program uses modified software from the classic comparative CT method (ΔΔCT) that takes multiple reference genes into account and uses inter-run calibration algorithms to correct for run-to-run differences [2, 3].

REFERENCES

1. Martens DS, Plusquin M, Gyselaers W, De Vivo I, Nawrot TS. Maternal pre-pregnancy body mass index and newborn telomere length. BMC Med. 2016; 14:148. https://doi.org/10.1186/s12916-016-0689-0 PMID:27751173

2. Grevendonk L, Janssen BG, Vanpoucke C, Lefebvre W, Hoxha M, Bollati V, Nawrot TS. Mitochondrial oxidative DNA damage and exposure to particulate air pollution in mother-newborn pairs. Environ Health. 2016; 15:10. https://doi.org/10.1186/s12940-016-0095-2 PMID:26792633

3. Hellemans J, Mortier G, De Paepe A, Speleman F, Vandesompele J. qBase relative quantification framework and software for management and automated analysis of real-time quantitative PCR data. Genome Biol. 2007; 8:R19. https://doi.org/10.1186/gb-2007-8-2-r19 PMID:17291332
