# Supplementary material for: The telomere-mitochondrial axis of aging in newborns
Source: Aging (Albany NY). 2022 Feb 15;14(4):1627–50. doi: 10.18632/aging.203897 (PMC8908926; doi:10.18632/aging.203897)
Supplement: Supplementary Figures [file aging-14-203897-s002.pdf]

SUPPLEMENTARY FIGURES

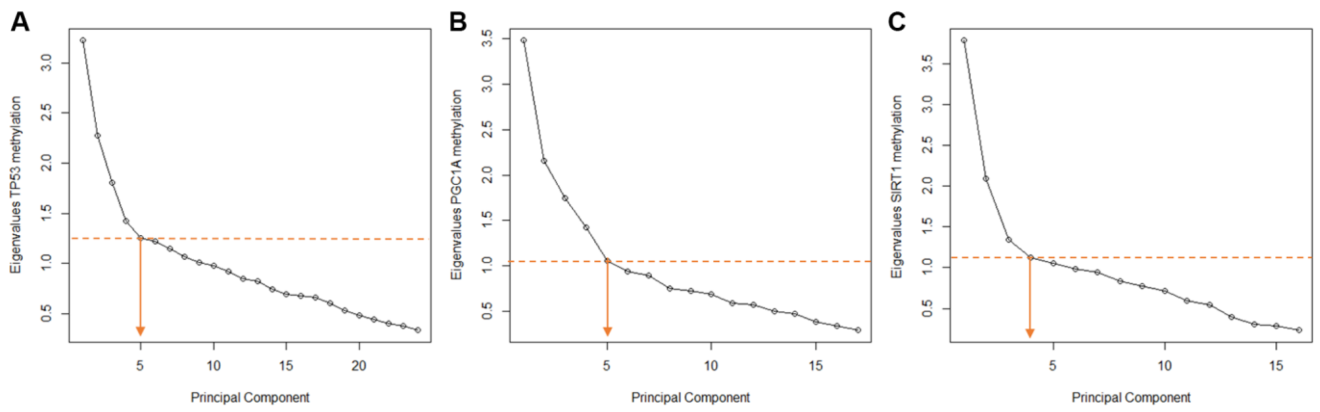

Supplementary Figure 1. Scree plots presenting eigenvalues of each Principal Component (PC). Eigenvalues represent the total amount of variance that can be explained by a given PC. Panel (**A**) displays the variance in *TP53* methylation explained by each PC. Panel (**B**) shows the variance in *PGC1A* methylation explained by each PC and panel (**C**) shows the variance in *SIRT1* methylation explained by each PC. Only PCs on the left side of the elbow in the curve were retained in the analysis.

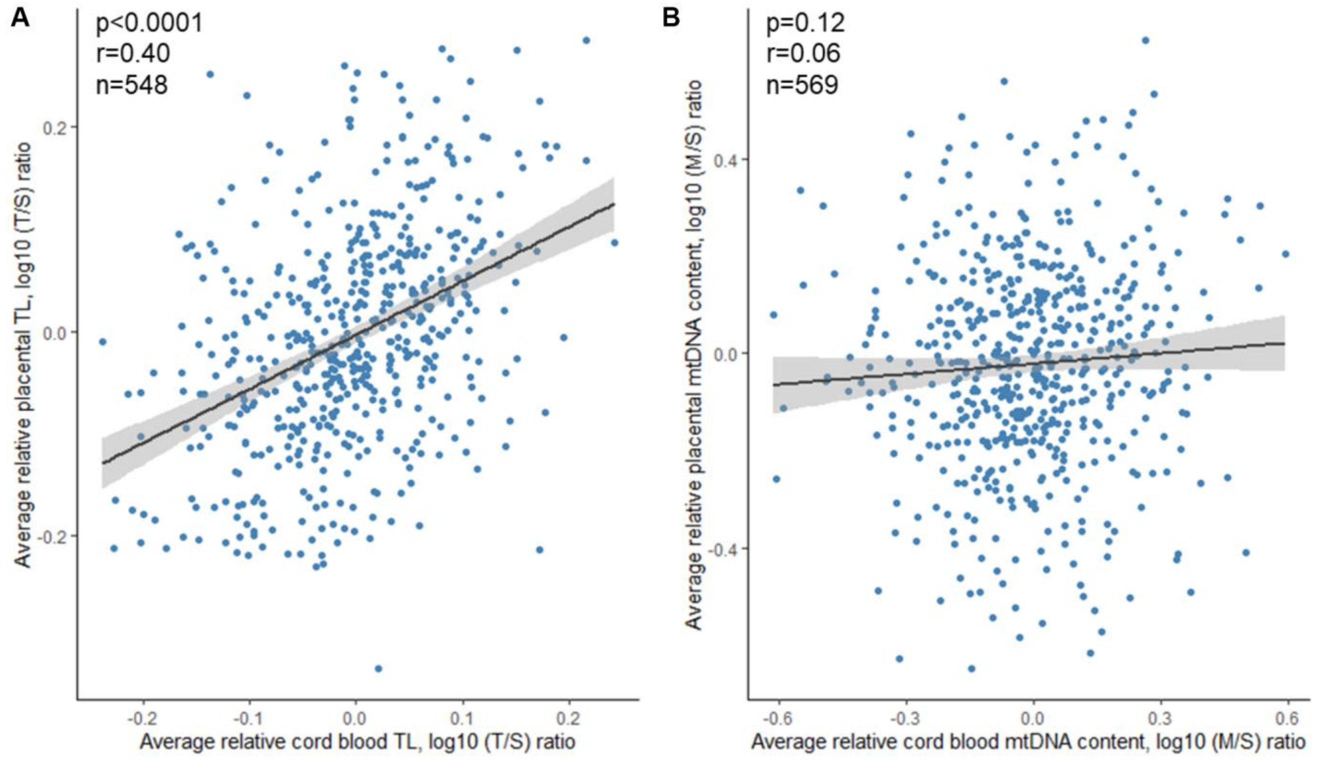

Supplementary Figure 2. Correlation between cord blood and placental age related markers. Panel (**A**) shows the correlation between average relative cord blood TL and average relative placental TL. Panel (**B**) shows the correlation between average relative cord blood mtDNAc and average relative placental mtDNAc. Abbreviations: TL: telomere length; T/S: telomere/single copy gene ratio; mtDNAc: mitochondrial DNA content; M/S: mitochondrial DNA/single copy gene ratio.

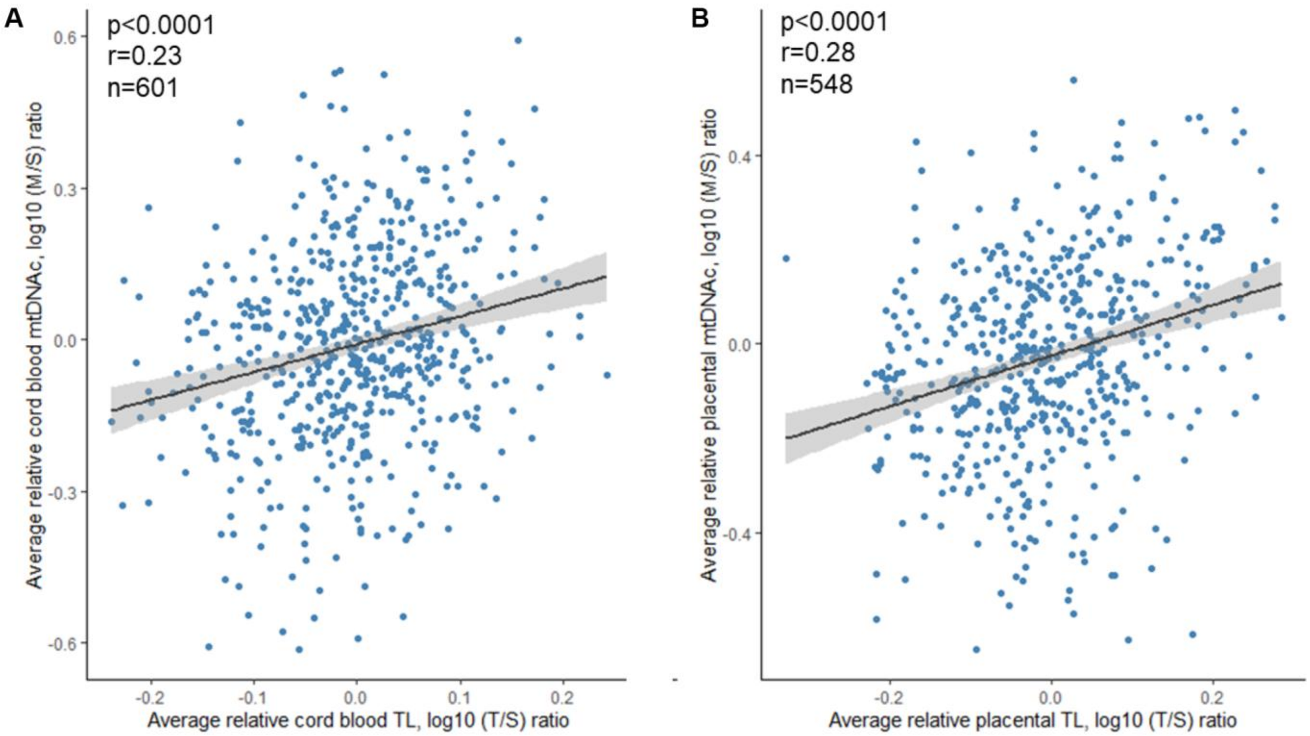

Supplementary Figure 3. Correlation between TL and mtDNAc. Panel (**A**) shows the correlation between average relative cord blood TL and mtDNAc. Panel (**B**) shows the correlation between average relative placental TL and mtDNAc. Abbreviations: TL: telomere length; T/S: telomere/single copy gene ratio; mtDNAc: mitochondrial DNA content; M/S: mitochondrial DNA/single copy gene ratio.

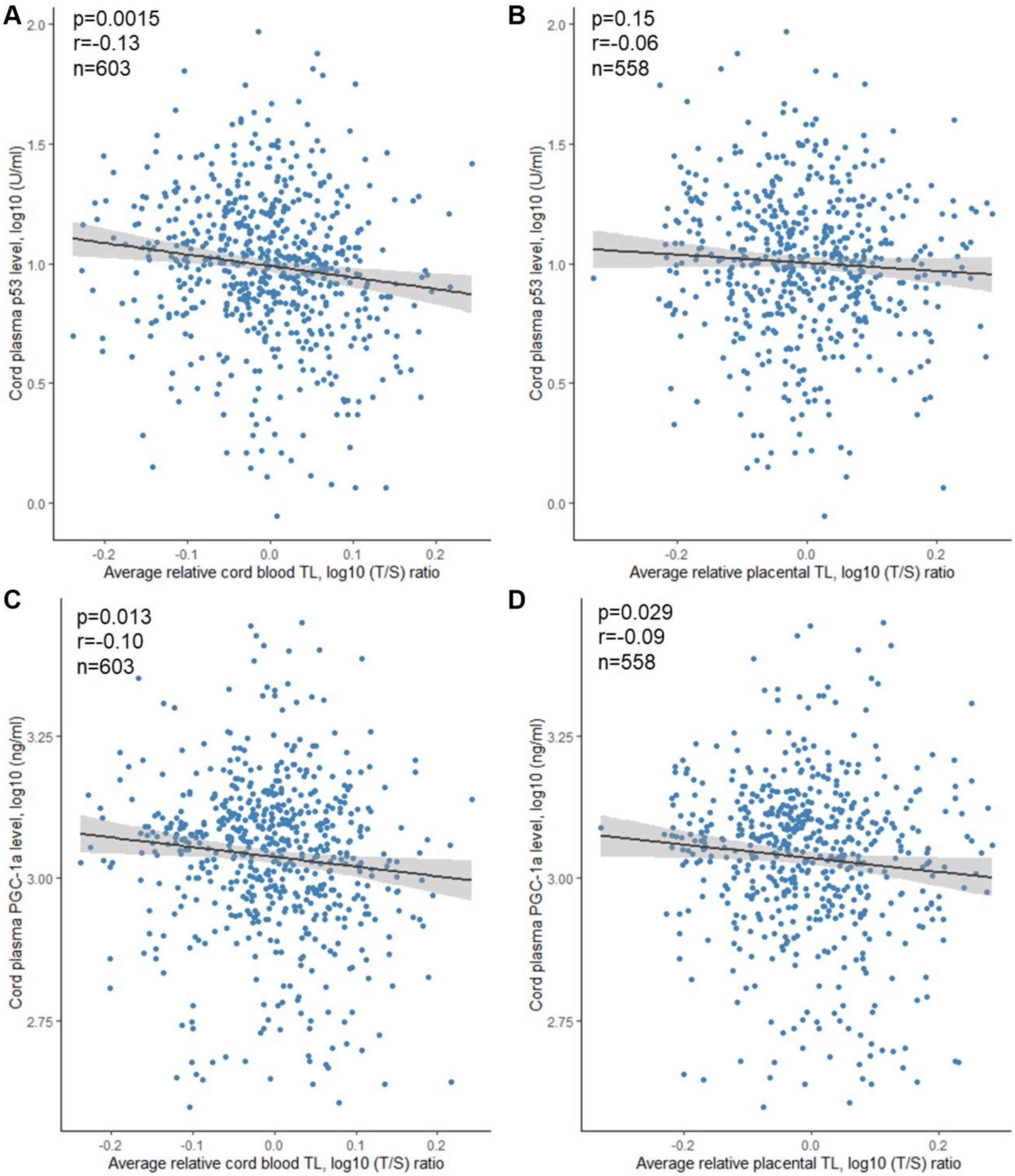

Supplementary Figure 4. Correlation between TL and cord plasma protein levels. Panel (**A**) shows the correlation between average relative cord blood TL and cord plasma p53 level. Panel (**B**) shows the correlation between average relative placental TL and cord plasma p53 level. Panel (**C**) shows the correlation between average relative cord blood TL and cord plasma PGC-1α level. Panel (**D**) shows the correlation between average relative placental TL and cord plasma PGC-1α level. Abbreviations: TL: telomere length; T/S: telomere/single copy gene ratio.

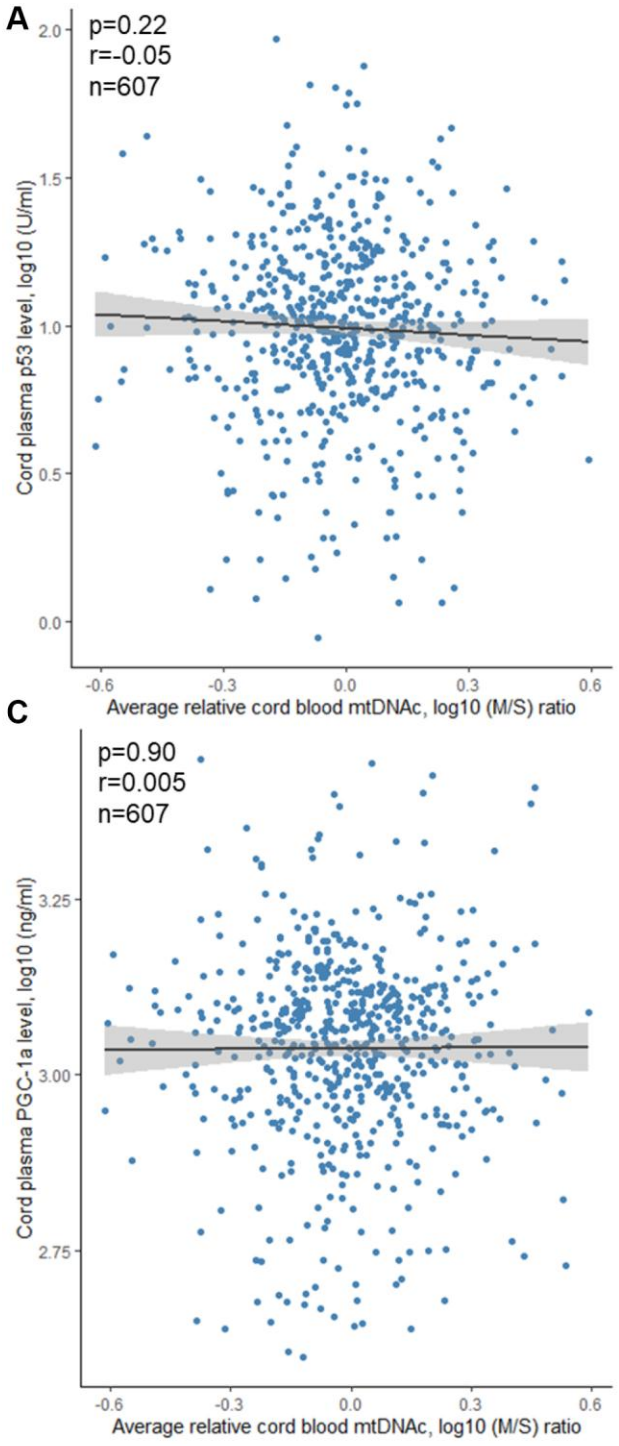

Supplementary Figure 5. Correlation between mtDNAc and cord plasma protein levels. Panel (**A**) shows the correlation between average relative cord blood mtDNAc and cord plasma p53 level. Panel (**B**) shows the correlation between average relative placental mtDNAc and cord plasma p53 level. Panel (**C**) shows the correlation between average relative cord blood mtDNAc and cord plasma PGC-1α level. Panel (**D**) shows the correlation between average relative placental mtDNAc and cord plasma PGC-1α level. Abbreviations: TL: telomere length; T/S: telomere/single copy gene ratio; mtDNAc: mitochondrial DNA content; M/S: mitochondrial DNA/single copy gene ratio.

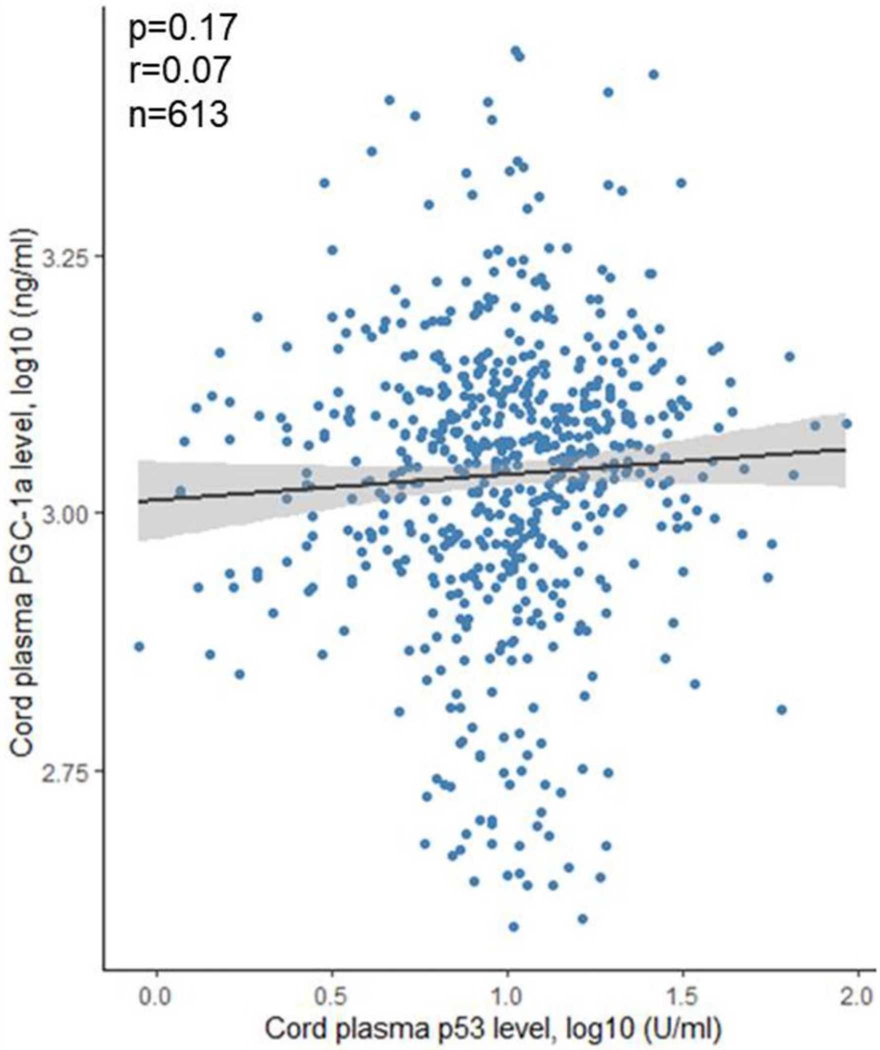

Supplementary Figure 6. Correlation between cord plasma p53 and cord plasma PGC-1α levels.

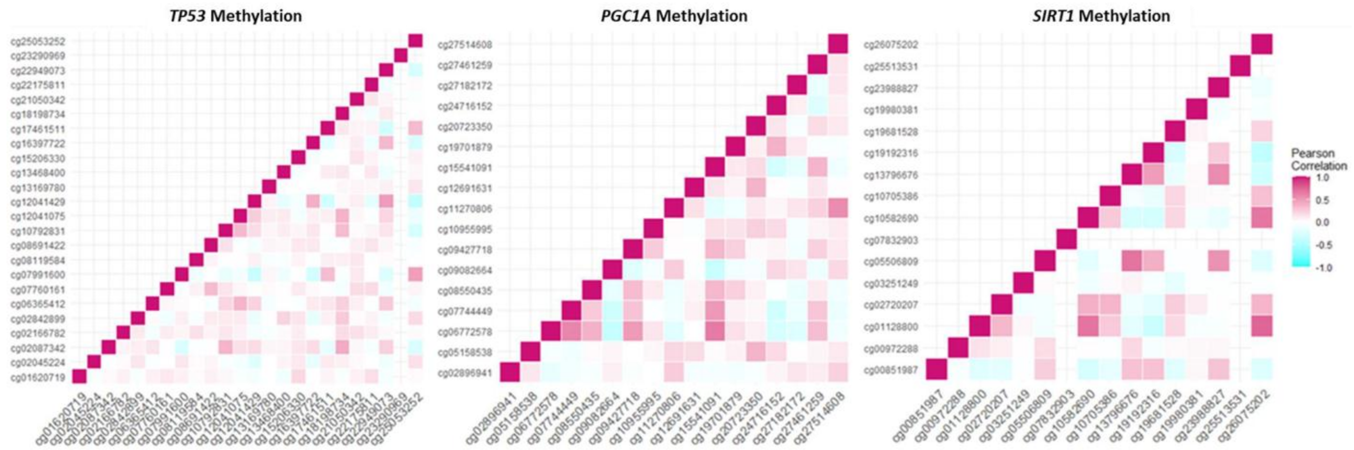

Supplementary Figure 7. Pearson correlation heatmap showing correlations between methylation levels of CpG regions for each gene separately. Methylation levels of *TP53*, *PGC1A* and *SIRT1* were obtained from the 850K array.

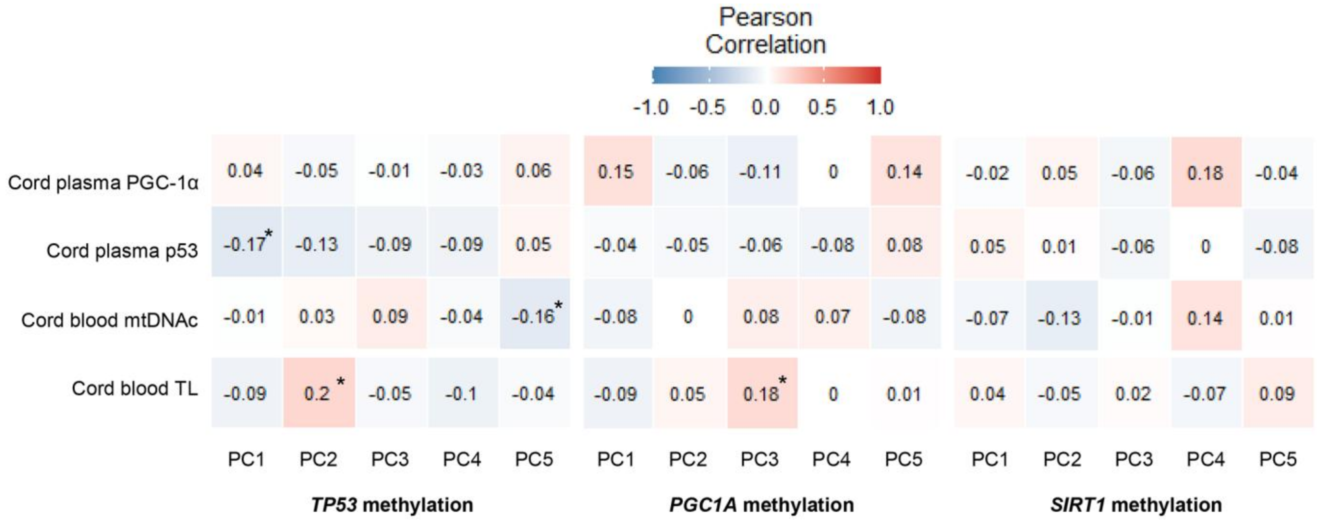

Supplementary Figure 8. Pearson correlation matrix showing correlations between age-related markers and methylation levels in cord blood. Methylation levels of *TP53*, *PGC1A* and *SIRT1* were obtained from the 850K array. All age-related markers are log-transformed. A star indicates *p*-values ≤0.05. Abbreviations: mtDNAc: Mitochondrial DNA content; TL: telomere length.

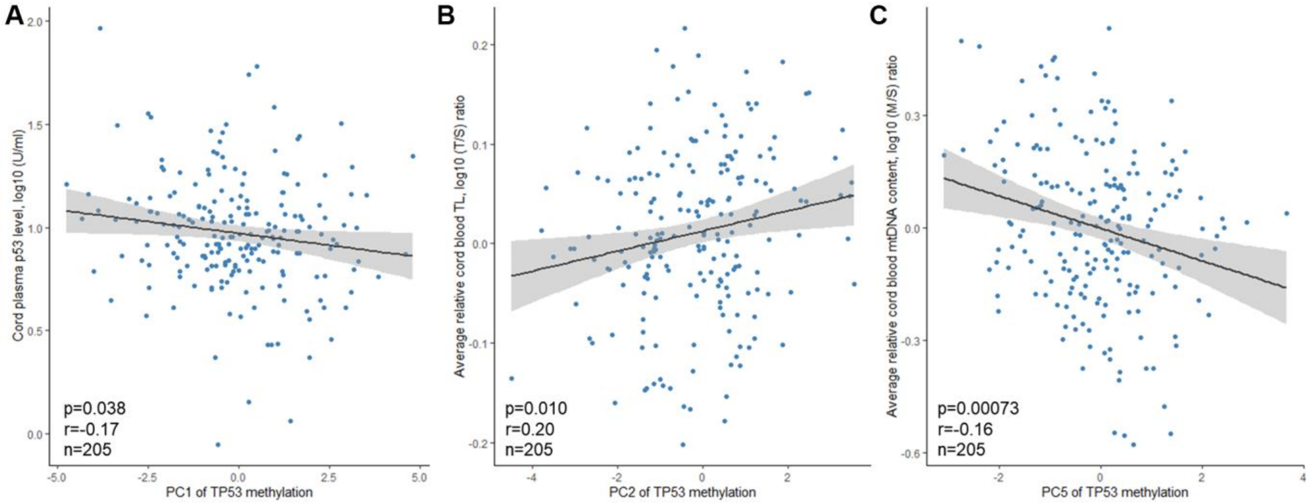

Supplementary Figure 9. Correlation between PCs of *TP53* methylation levels and cord blood age-related markers. Panel (**A**) shows a negative correlation between PC1 and cord plasma p53 level. Panel (**B**) shows a positive correlation between PC2 and average relative cord blood TL. Panel (**C**) shows a negative correlation between PC5 and average relative cord blood mtDNA content. Abbreviations: TL: telomere length; T/S: telomere/single copy gene ratio; mtDNAc: Mitochondrial DNA content; M/S: Mitochondrial DNA/single copy gene ratio.

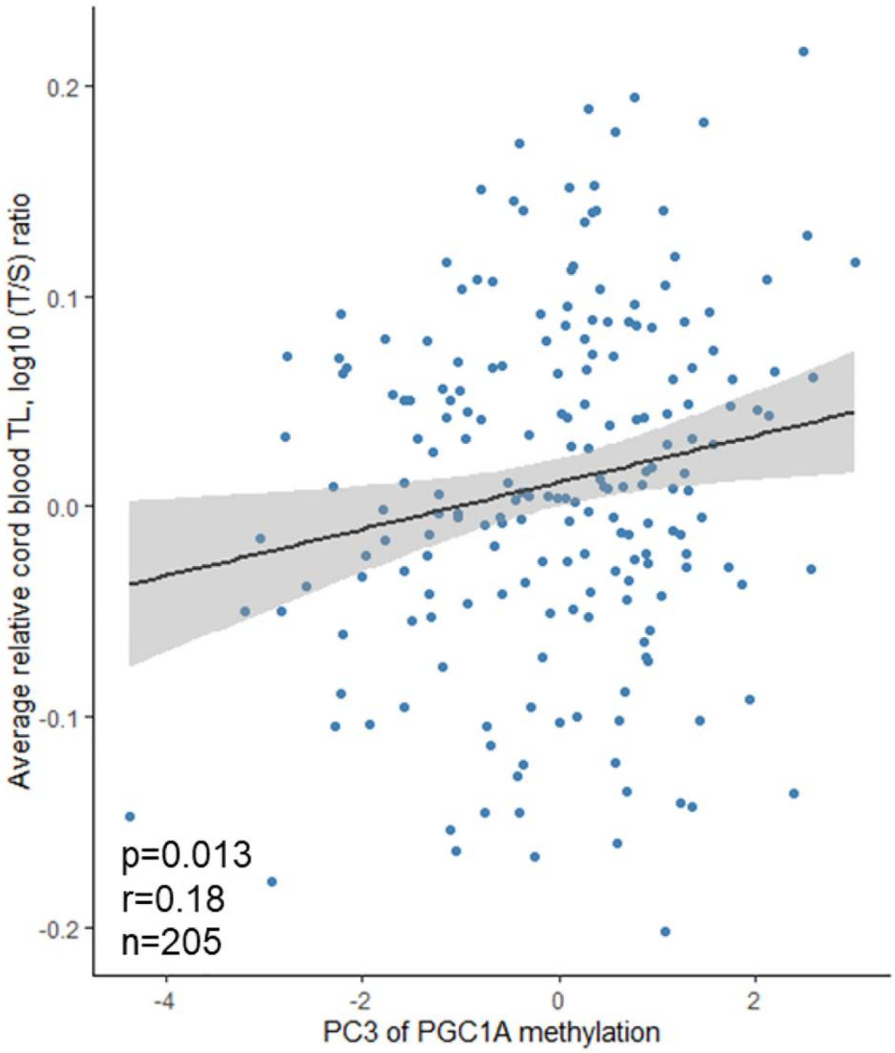

Supplementary Figure 10. Correlation between PCs of *PGC1A* methylation levels and cord blood TL. A positive correlation is shown between PC3 and average relative cord blood TL. Abbreviations: TL: telomere length; T/S: telomere/single copy gene ratio.
