# Supplementary material for: The telomere-mitochondrial axis of aging in newborns
Source: Aging (Albany NY). 2022 Feb 15;14(4):1627–50. doi: 10.18632/aging.203897 (PMC8908926; doi:10.18632/aging.203897)
Supplement: Supplementary Tables [file aging-14-203897-s003.pdf]

SUPPLEMENTARY TABLES

Supplementary Table 1. 850K methylation information. Annotations were obtained using the BiocManager package in R.

850K methylation array							
**CpG Name**	**Annotation**	**Position**	**Relation to Island**	**UCSC Ref Gene Name**	**UCSC Ref Gene Accession**	**UCSC Ref Gene Group**	**Regulatory Feature Group**
cg25053252	chr17:7589290-7589503	7589358	Island	***TP53***	NM_000546	5′UTR	PA
cg17461511	chr17:7591494-7591839	7591552	Island	***TP53***	NM_000546	TSS1500	PA
cg02045224	chr17:7591494-7591839	7591618	Island	***TP53***	NM_000546	TSS1500	PA
cg07991600	chr17:7589290-7589503	7589380	Island	***TP53***	NM_000546	5′UTR	PA
cg02087342	NA	7579047	Open Sea	***TP53***	NM_000546	Body	
cg22949073	NA	7572391	Open Sea	***TP53***	NM_000546	3′UTR	
cg13169780	chr17:7591494-7591839	7591570	Island	***TP53***	NM_000546	TSS1500	PA
cg07760161	chr17:7589290-7589503	7588378	N Shore	***TP53***	NM_000546	5′UTR	PA; CTS
cg02166782	chr17:7591494-7591839	7591564	Island	***TP53***	NM_000546	TSS1500	PA
cg22175811	chr17:7591494-7591839	7590052	N Shore	***TP53***	NM_000546	5′UTR	PA
cg21050342	chr17:7591494-7591839	7591644	Island	***TP53***	NM_000546	TSS1500	PA
cg13468400	NA	7579546	Open Sea	***TP53***	NM_000546	Body	
cg08119584	chr17:7591494-7591839	7589976	N Shore	***TP53***	NM_000546	5′UTR	PA
**cg18198734**	**NA**	**7579263**	**Open Sea**	***TP53***	**NM_000546**	**Body**	
cg23290969	chr17:7589290-7589503	7589272	N Shore	***TP53***	NM_000546	5′UTR	PA
cg16397722	NA	7577090	Open Sea	***TP53***	NM_000546	Body	
cg12041075	NA	7579312	Open Sea	***TP53***	NM_000546	Body	
cg08691422	chr17:7589290-7589503	7588787	N Shore	***TP53***	NM_000546	5′UTR	
cg15206330	chr17:7591494-7591839	7591591	Island	***TP53***	NM_000546	TSS1500	PA
**cg12041429**	**chr17:7589290-7589503**	**7585875**	**N Shelf**	***TP53***	**NM_000546**	**5′UTR**	
cg02842899	chr17:7589290-7589503	7589491	Island	***TP53***	NM_000546	5′UTR	PA
cg01620719	chr17:7589290-7589503	7589251	N Shore	***TP53***	NM_000546	5′UTR	PA
cg10792831	NA	7578689	Open Sea	***TP53***	NM_000546	Body	
cg06365412	NA	7580709	Open Sea	***TP53***	NM_000546	5′UTR	
cg27182172	chr4:23890120-23890955	23890890	Open Sea	***PGC1A***	NM_013261	Body	
cg09082664	chr4:23499647-23499989	23890701	Open Sea	***PGC1A***	NM_013261	Body	
cg27514608	chr4:23892305-23892675	23892540	Open Sea	***PGC1A***	NM_013261	TSS1500	
cg05158538	chr4:23891440-23891870	23891486	Open Sea	***PGC1A***	NM_013261	Body	
cg10955995	chr4:23857865-23858250	23858146	Open Sea	***PGC1A***	NM_013261	Body	
cg27461259	chr4:23892305-23892675	23892621	Open Sea	***PGC1A***	NM_013261	TSS1500	
cg07744449	NA	23887314	Open Sea	***PGC1A***	NM_013261	Body	
cg08550435	chr4:23829600-23829910	23829879	Open Sea	***PGC1A***	NM_013261	Body	
cg02896941	chr4:23499647-23499989	23890659	Open Sea	***PGC1A***	NM_013261	Body	
cg19701879	chr4:23880820-23881155	23881105	Open Sea	***PGC1A***	NM_013261	Body	
cg11270806	chr4:23892305-23892675	23892515	Open Sea	***PGC1A***	NM_013261	TSS1500	

cg24716152	chr4:23879168-23881165	23879965	Open Sea	***PGC1A***	NM_013261	Body	
cg12691631	chr4:23891440-23891870	23891835	Open Sea	***PGC1A***	NM_013261	TSS200	
cg15541091	chr4:23812780-23813175	23813125	Open Sea	***PGC1A***	NM_013261	Body	
cg06772578	NA	23796624	Open Sea	***PGC1A***	NM_013261	3′UTR	
cg09427718	chr4:23819243-23820433	23819929	Open Sea	***PGC1A***	NM_013261	Body	
cg20723350	chr4:23891440-23891870	23891596	Open Sea	***PGC1A***	NM_013261;<br>NM_013261	1stExon;<br>5′UTR	
cg10582690	chr10:69644169-69645178	69644422	Island	***SIRT1***	NM_012238	TSS200	PA
cg07832903	chr10:69644169-69645178	69644926	Island	***SIRT1***	NM_012238	Body	PA
cg00972288	chr10:69644169-69645178	69644348	Island	***SIRT1***	NM_012238	TSS200	PA
cg19681528	chr10:69644169-69645178	69644405	Island	***SIRT1***	NM_012238	TSS200	PA
cg02720207	chr10:69644169-69645178	69644399	Island	***SIRT1***	NM_012238	TSS200	PA
cg25513531	chr10:69644169-69645178	69644407	Island	***SIRT1***	NM_012238	TSS200	PA
cg26075202	chr10:69644169-69645178	69645177	Island	***SIRT1***	NM_012238	Body	
cg10705386	chr10:69644169-69645178	69644512	Island	***SIRT1***	NM_012238	1stExon	PA
cg01128800	chr10:69644169-69645178	69644938	Island	***SIRT1***	NM_012238	Body	PA
cg00851987	chr10:69662340-69664884	69664037	Open Sea	***SIRT1***	NM_012238	Body	
cg03251249	chr10:69644169-69645178	69645012	Island	***SIRT1***	NM_012238	Body	PA
cg05506809	chr10:69644169-69645178	69648299	S Shelf	***SIRT1***	NM_012238	Body	
cg19192316	chr10:69644169-69645178	69643020	N Shore	***SIRT1***	NM_012238	TSS1500	
cg23988827	NA	69676715	Open Sea	***SIRT1***	NM_012238	3'UTR	
cg19980381	chr10:69644169-69645178	69644128	N Shore	***SIRT1***	NM_012238	TSS1500	PA
cg13796676	chr10:69644169-69645178	69648687	S Shelf	***SIRT1***	NM_012238	Body	

Abbreviations: NA: not applicable; PA: Promotor Associated; CTS: Cell Type Specific.

Supplementary Table 2. Rotated loadings. Bold loadings have an absolute value larger than 0.45, meaning the CpG is selected as relevant for the specific PC.

			TP53		
**CpG**	**PC1**	**PC2**	**PC3**	**PC4**	**PC5**
cg01620719	–0.11	0.16	0.35	0.02	0.44
cg02045224	–0.20	0.27	–0.07	0.17	–0.16
cg02087342	**0.55**	0.27	0.06	–0.08	–0.16
cg02166782	–0.01	0.32	0.31	0.27	–0.26
cg02842899	–0.12	**0.47**	–0.05	0.04	–0.35
cg06365412	0.37	0.36	–0.27	–0.06	0.42
cg07760161	–0.01	0.18	**0.60**	–0.25	0.25
cg07991600	**–0.56**	0.26	–0.22	0.20	0.32
cg08119584	0.03	0.16	0.18	0.34	–0.23
cg08691422	0.04	0.21	**0.58**	–0.16	0.04
cg10792831	**0.49**	0.42	0.14	–0.32	0.03
cg12041075	0.43	**0.46**	–0.24	–0.15	0.09
cg12041429	**0.72**	–0.25	–0.02	0.24	0.09
cg13169780	0.04	0.14	0.04	0.26	0.03
cg13468400	–0.04	0.32	0.04	–0.44	–0.36

cg15206330	–0.07	0.23	0.36	0.20	–0.03
cg16397722	**0.54**	–0.21	0.17	0.00	0.03
cg17461511	–0.42	0.44	–0.25	–0.15	0.13
cg18198734	0.35	**0.61**	–0.25	–0.06	–0.21
cg21050342	0.05	0.25	0.31	**0.47**	0.25
cg22175811	–0.17	0.17	0.38	–0.06	0.33
cg22949073	**0.64**	–0.10	–0.05	0.34	0.07
cg23290969	–0.03	0.20	0.22	0.00	–0.40
cg25053252	**–0.62**	0.29	–0.19	0.10	–0.01

PGC1A

CpG	PC1	PC2	PC3	PC4	PC5
cg02896941	0.04	0.06	0.02	–0.07	0.02
cg05158538	0.08	0.07	–0.09	–0.10	–0.06
cg06772578	0.30	0.21	0.21	–0.12	–0.07
cg07744449	0.20	0.19	0.19	–0.09	–0.09
cg08550435	**0.45**	0.05	0.10	–0.08	–0.11
cg09082664	–0.04	–0.14	–0.14	0.07	–0.11
cg09427718	**0.54**	–0.05	0.10	0.06	0.08
cg10955995	0.20	–0.02	0.10	0.08	0.05
cg11270806	0.04	–0.02	–0.05	–0.08	0.04
cg12691631	0.06	0.03	–0.02	0.04	–0.16
cg15541091	0.20	0.32	0.20	–0.11	–0.19
cg19701879	**0.48**	–0.13	–0.14	0.06	–0.02
cg20723350	0.05	0.03	0.00	0.02	–0.09
cg24716152	0.29	–0.26	–0.22	0.14	0.02
cg27182172	–0.02	–0.07	–0.09	–0.01	0.03
cg27461259	0.04	0.12	0.01	–0.01	0.03
cg27514608	0.20	–0.11	–0.01	0.00	–0.08

SIRT1

CpG	PC1	PC2	PC3	PC4	PC5
cg00851987	**–0.51**	0.14	–0.10	–0.15	0.28
cg00972288	–0.01	0.28	–0.08	**0.68**	–0.22
cg01128800	**0.69**	**0.45**	–0.32	–0.08	–0.16
cg02720207	**0.49**	0.40	0.18	–0.17	0.04
cg03251249	0.00	–0.03	–0.22	**0.59**	**0.48**
cg05506809	**–0.56**	**0.60**	–0.12	0.13	–0.06
cg07832903	0.01	0.00	**0.55**	0.27	**–0.62**
cg10582690	**0.73**	0.34	–0.15	–0.21	–0.16
cg10705386	0.41	0.36	0.19	0.02	0.11
cg13796676	**–0.66**	**0.57**	–0.05	0.21	–0.01
cg19192316	**–0.65**	0.18	0.03	–0.07	0.25
cg19681528	0.35	0.23	0.25	–0.24	–0.08
cg19980381	–0.02	0.05	**0.64**	0.03	0.28
cg23988827	**–0.46**	**0.61**	–0.17	–0.09	–0.13
cg25513531	0.08	0.35	0.42	0.11	0.03
cg26075202	**0.76**	0.31	–0.27	–0.21	–0.14

Supplementary Table 3. Comparison of the characteristics between our study population (*n* = 613) and full ENVIR*ON*AGE data (*n* = 1530).

Characteristic	Analyzed population Mean ± SD or *n* (%) (*n* = 613)	Total population Mean ± SD or *n* (%) (*n* = 1530)	*P*-value
**Mothers**			
Age, y	29.3 ± 4.6	29.4 ± 4.55	0.64
Pre-pregnancy BMI, kg/m^2	24.6 ± 4.8	24.5 ± 4.76	0.66
Educational level			
Low	79 (12.9%)	160 (10.5%)	0.13
Middle	227 (37.0%)	500 (32.7%)	0.064
High	307 (50.1%)	726 (47.5%)	0.32
Smoking status			
Never smoker	391 (63.8%)	897 (58.6%)	0.030
Former smoker	154 (25.1%)	326 (21.3%)	0.064
Current smoker	68 (11.1%)	173 (11.3%)	0.95
Parity			
1	337 (55.0%)	751 (49.1%)	0.015
2	206 (33.6%)	507 (33.1%)	0.86
≥	70 (11.4%)	172 (11.2%)	0.95
**Newborns**			
Sex			
Female	321 (52.4%)	736 (48.1%)	0.10
Gestational age, wk	39.2 ± 1.7	39.2 ± 1.6	0.91
Birth weight, g	3420 ± 496	3400 ± 495	0.52
Ethnicity			
European-Caucasian	533 (86.9%)	1221 (79.8%)	0.0001
Season of birth			
Winter	150 (24.5%)	372 (24.3%)	0.97
Spring	149 (24.3%)	344 (22.5%)	0.40
Summer	151 (24.6%)	329 (21.5%)	0.13
Autumn	163 (26.6%)	385 (25.2%)	0.54

Supplementary Table 4. Association between mitochondrial DNA content (mtDNAc) and cord plasma protein levels (p53 and PGC-1α) and between the cord plasma protein levels.

	Cord p53 (*n* = 613)		Cord PGC-1α (*n* = 607)	
	% difference (95% CI)	*P*-value	% difference (95% CI)	*P*-value
**Cord mtDNAc**	–0.0082 (–0.51, 0.50)	0.99	0.27 (–0.91, 1.47)	0.26
**Placenta mtDNAc***	–0.30 (–0.86, 0.27)	0.31	–0.74 (–2.05, 0.58)	0.27
**Cord PGC-1α**	0.12 (–0.21, 0.44)	0.51	NA	NA

Estimates are presented as percentage difference with 95% CI for a 10% change in explanatory variable. All models are adjusted for technical covariates (sample storage and batch effects), newborn's sex, gestational age, maternal BMI, maternal and paternal age, ethnicity, parity, smoke status, maternal education and month of delivery. Cord plasma p53 and PGC-1α represent the exposure variable, while the variables in the left column represent the outcome variable. *Data available for *n* = 575. Abbreviation: NA: not applicable.

Supplementary Table 5. Association between Principle Components (PCs) of *TP53*, *PGC1A* and *SIRT1* methylation levels and cord blood telomere length, cord blood mitochondrial DNA content (mtDNAc) and cord plasma protein levels (p53 and PGC-1α).

	Cord TL (*n* = 200)	Cord mtDNA (*n* = 205)	Cord p53 (*n* = 205)	Cord PGC-1α (*n* = 205)
	% difference (95% CI)	% difference (95% CI)	% difference (95% CI)	% difference (95% CI)
TP53				
PC1	−1.00 (−2.48, 0.50)	−0.48 (−4.43, 3.63)	**−5.58 (−1.25, −0.66)**	0.27 (−2.72, 3.35)
PC2	**1.74 (−0.073, 3.58)**	0.32 (−4.51, 5.39)	**−4.63 (−9.79, 0.83)**	−1.00 (−4.55, 2.68)
PC3	−1.38 (−3.36, 0.65)	−0.11 (−5.62, 5.71)	−3.23 (−9.73, 3.74)	0.13 (−3.90, 4.33)
PC4	**−2.46 (−4.84, -0.024)**	−2.96 (−9.28, 3.81)	−4.63 (−12.42, 3.85)	−0.20 (−5.10, 4.96)
PC5	−0.63 (−2.84, 1.64)	**−10.52 (−15.59,-5.14)**	2.59 (−5.16, 10.96)	0.77 (−3.79, 5.54)
PGC1A				
PC1	−0.31 (−1.88, 1.29)	−0.17 (4.06, 3.89)	−2.51 (7.33, 2.55)	1.15 (−1.82, 4.22)
PC2	−0.65 (-2.47, 1.21)	−0.34 (−5.24, 4.81)	−1.39 (−7.30, 4.89)	−0.26 (−3.82, 3.44)
PC3	**2.20 (0.11, 4.34)**	4.47 (−0.92, 10.78)	−0.18 (−7.08, 7.024)	−0.77 (−4.87, 3.52)
PC4	−0.77 (-2.99, 1.50)	2.50 (−3.38, 8.74)	−3.87 (−11.09, 3.93)	0.98 (−3.57, 5.74)
PC5	0.91 (-1.66, 3.54)	−3.98 (−10.38, 2.88)	3.58 (−5.27, 13.25)	2.71 (−2.55, 8.25)
SIRT1				
PC1	0.12 (-1.27, 1.52)	−2.43 (−5.96, 1.32)	0.73 (−3.80, 5.47)	−0.26 (−2.93, 2.48)
PC2	−0.41 (-2.25, 1.46)	**−4.15 (−8.42, 0.80)**	−0.76 (−6.84, 5.72)	−0.79 (−4.42, 2.97)
PC3	1.00 (-1.31, 3.37)	−1.18 (−6.97, 4.96)	−3.23 (−10.66, 4.81)	−2.57 (−7.04, 2.12)
PC4	0.52 (-1.99, 3.08)	4.20 (−2.30, 11.14)	0.48 (−7.79, 9.48)	4.41 (−0.70, 9.78)
PC5	1.10 (-1.63, 3.91)	0.11 (−6.92, 7.67)	−5.28 (−13.83, 4.12)	−0.77 (−6.16, 4.94)

Estimates are presented as percentage difference with 95% CI for a one-unit change in exposure variable. All models are adjusted for technical covariates (sample storage and batch effects), newborn's sex, gestational age, maternal BMI, maternal and paternal age, ethnicity, parity, smoke status, maternal education and month of delivery. The variables in columns represent the response variables, while the variables in the rows represent the explanatory variables. Bold values indicate significant estimates.
